# Supplementary figures and images for: Clinical significance of anti-NT5c1A autoantibody in Korean patients with inflammatory myopathies
Source: PLoS One. 2023 Apr 14;18(4):e0284409. doi: 10.1371/journal.pone.0284409 (PMC10104319; doi:10.1371/journal.pone.0284409)

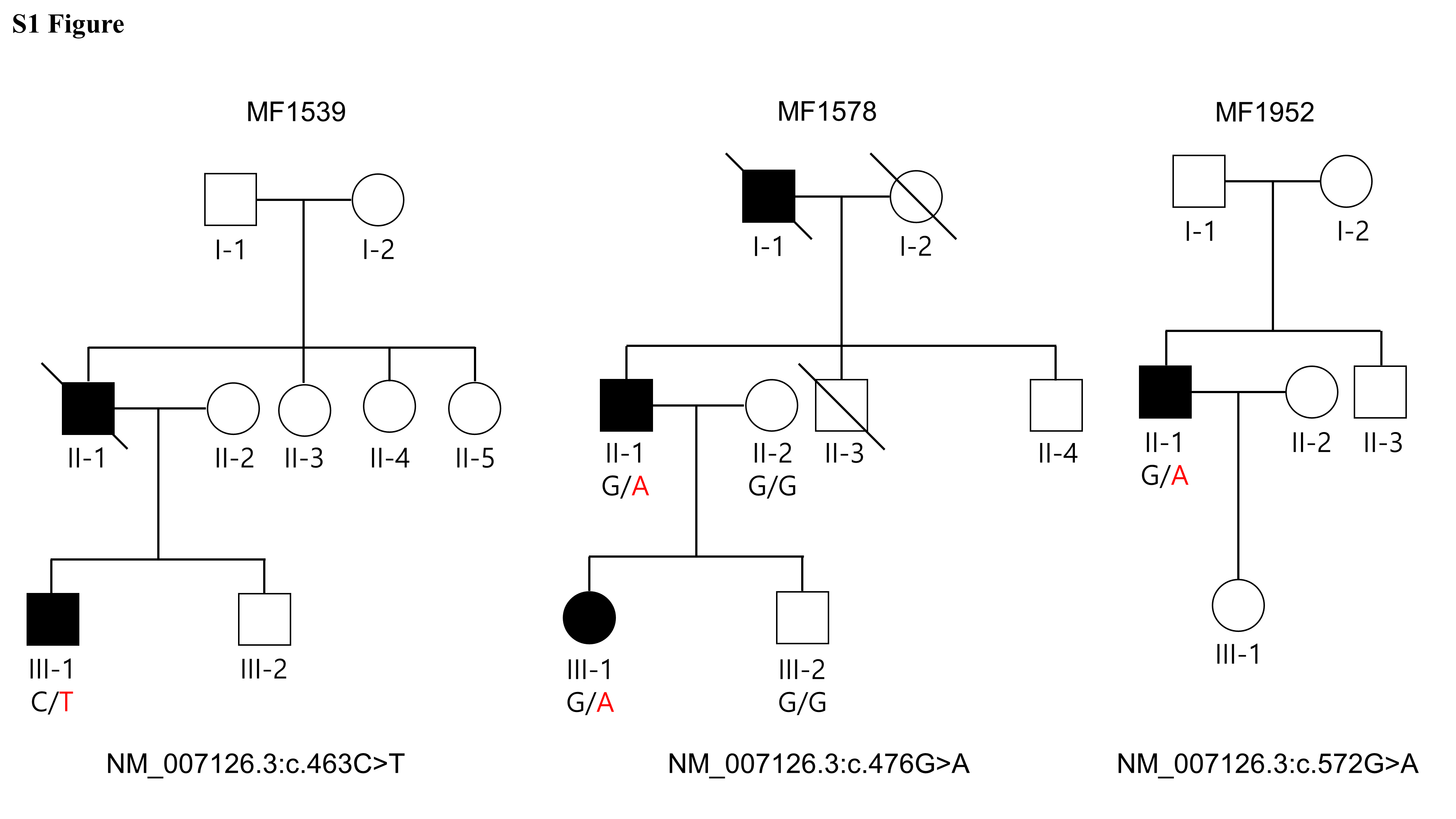

Supplement: S1 Fig — (TIF) [file pone.0284409.s001.TIF]
