# Supplementary material for: Clinical significance of anti-NT5c1A autoantibody in Korean patients with inflammatory myopathies
Source: PLoS One. 2023 Apr 14;18(4):e0284409. doi: 10.1371/journal.pone.0284409 (PMC10104319; doi:10.1371/journal.pone.0284409)
Supplement: S1 Table — (DOCX) [file pone.0284409.s002.docx]

S1 Table. Reactivity of myositis-specific autoantibodies in sera from patients with IBM

| Myositis-specific autoantibodies | All IBM patients (n=20) | | anti-NT5c1A positive (n=8) | |
| --- | --- | --- | --- | --- |
|  | n | Frequency | n | frequency |
| Anti-Ro52 | 4 | 20.0% | 2 | 25.0% |
| Anti-SRP | 1 | 5.0% | 1 | 1.3% |
| Anti-MDA5 | 1 | 5.0% | 1 | 1.3% |
| Anti-SAE1 | 1 | 5.0% | 1 | 1.3% |
| Anti-Mi-2β | 1 | 5.0% | 0 | 0.0% |
| Anti-Mi-2α | 0 | 0.0% | 0 | 0.0% |
| Anti-TIF1γ | 0 | 0.0% | 0 | 0.0% |
| Anti-NXP2 | 0 | 0.0% | 0 | 0.0% |
| Anti-Ku | 0 | 0.0% | 0 | 0.0% |
| Anti-PM-Scl100 | 0 | 0.0% | 0 | 0.0% |
| Anti-PM-Scl75 | 0 | 0.0% | 0 | 0.0% |
| Anti-Jo-1 | 0 | 0.0% | 0 | 0.0% |
| Anti-PL-7 | 0 | 0.0% | 0 | 0.0% |
| Anti-PL-12 | 0 | 0.0% | 0 | 0.0% |
| Anti-EJ | 0 | 0.0% | 0 | 0.0% |
| Anti-OJ | 0 | 0.0% | 0 | 0.0% |
