# Supplementary material for: Clinical significance of anti-NT5c1A autoantibody in Korean patients with inflammatory myopathies
Source: PLoS One. 2023 Apr 14;18(4):e0284409. doi: 10.1371/journal.pone.0284409 (PMC10104319; doi:10.1371/journal.pone.0284409)
Supplement: S2 Table — (DOCX) [file pone.0284409.s003.docx]

S2 Table. Clinical features of 103 inflammatory myopathy patients according to anti-NT5c1A antibody status

|  | Patients with inflammatory myopathies (n=103) | | |
| --- | --- | --- | --- |
|  | anti-NT5c1A positive (n=13) | anti-NT5c1A negative (n=90) | p-value |
| Male | 7 (54) | 33 (37) | 0.377 |
| Age at symptom onset (Y) | 52 [40 – 57] | 56 [46 – 64] | 0.509 |
| Age at diagnosis (Y) | 59 [46 – 61] | 57 [48 – 65] | 0.837 |
| Disease duration (M) | 24 [3 – 36] | 10 [3 – 26] | 0.348 |
| Serum CK (IU/l) | 581 [259 – 1042] | 2,921 [716-7801] | 0.019 |
| Dysphagia | 3 (23) | 19 (21) | 1.000 |
| Skin rash | 3 (23) | 12 (13) | 0.689 |
| Other antibodies* | 8 (62) | 34 (38) | 0.184 |

Note: values are expressed as number (%) or median [interquartile range]. Y, years; M, months; CK, creatine kinase. *Antibodies against Mi-2α, Mi-2β, TIF1γ, MDA5, NXP2, SAE1, Ku, PM-Scl100, PM-Scl75, Jo-1, SRP, PL-7, PL-12, EJ, OJ, and Ro-52 were included.
