# Supplementary material for: Clinical significance of anti-NT5c1A autoantibody in Korean patients with inflammatory myopathies
Source: PLoS One. 2023 Apr 14;18(4):e0284409. doi: 10.1371/journal.pone.0284409 (PMC10104319; doi:10.1371/journal.pone.0284409)
Supplement: S3 Table — (DOCX) [file pone.0284409.s004.docx]

S3 Table. Clinical features of patients with inflammatory myopathies other than IBM according to anti-NT5c1A antibody status

|  | Patients with inflammatory myopathies other than IBM (n=83) | | |
| --- | --- | --- | --- |
|  | anti-NT5c1A positive (n=5) | anti- NT5c1A negative (n=78) | p-value |
| Male | 2 (40) | 28 (36) | 1.000 |
| Age at symptom onset (Y) | 44 [39 – 60] | 55 [44 – 63] | 0.640 |
| Age at diagnosis (Y) | 46 [39 – 60] | 56 [44 – 64] | 0.516 |
| Disease duration (M) | 1 [1 – 3] | 8 [3 – 12] | 0.052 |
| Serum CK (IU/L) | 2,125 [43 – 9952] | 4,171 [1151-8415] | 0.411 |
| Dysphagia | 0 (0) | 13 (17) | 0.575 |
| Skin rash | 2 (40) | 12 (15) | 0.272 |
| Other antibodies* | 4 (80) | 31 (40) | 0.157 |

Note: values are expressed as number (%) or median [interquartile range]. Y, years; M, months; CK, creatine kinase. *Antibodies against Mi-2α, Mi-2β, TIF1γ, MDA5, NXP2, SAE1, Ku, PM-Scl100, PM-Scl75, Jo-1, SRP, PL-7, PL-12, EJ, OJ, and Ro-52 were included.
